# Genotyping and Characterization of HPV Status, Hypoxia, and Radiosensitivity in 22 Head and Neck Cancer Cell Lines

**DOI:** 10.3390/cancers13051069

**Published:** 2021-03-03

**Authors:** Eva-Leonne Göttgens, Marleen Ansems, William P. J. Leenders, Johan Bussink, Paul N. Span

**Affiliations:** 1 Radiotherapy and OncoImmunology Laboratory, Department of Radiation Oncology, Radboud University Medical Center, 6525 GA Nijmegen, The Netherlands; EGottgens@amphia.nl (E.-L.G.); marleen.ansems@radboudumc.nl (M.A.); jan.bussink@radboudumc.nl (J.B.); 2 Department of Clinical Chemistry and Hematology, Amphia Hospital, 4818 CK Breda, The Netherlands; 3 Department of Biochemistry, Radboud Institute for Molecular Life Sciences, University Medical Centre, 6525 GA Nijmegen, The Netherlands; William.Leenders@radboudumc.nl

**Keywords:** head and neck squamous cell carcinoma, mutations, radiosensitivity

## Abstract

**Simple Summary:**

Cell lines are widely used for research, but even commonly used cell lines may not be well characterized, making validation of published data and generalizing the results to the clinic problematic. Here, we have established a head and neck squamous cell carcinomas (HNSCC) cell line database. We believe that these cell lines are suitable models to study HNSCC disease in vitro and in vivo and may provide a valuable tool for investigators wishing to study the relation between e.g., hypoxia and radiosensitivity in HNSCC.

**Abstract:**

To study head and neck squamous cell carcinomas (HNSCC) in vitro, a large variety of HNSCC cell lines have been developed. Here, we characterize a panel of 22 HNSCC cell lines, thereby providing a tool for research into tumor-specific treatment options in HNSCC. Both human papillomavirus (HPV) positive and HPV negative tumor cell lines were collected from commercial and collaborative sources. Short tandem repeat profiling was used to confirm or characterize the identity of the cell lines. Targeted sequencing was performed using a standard pathology single molecule Molecular Inversion Probe panel to detect mutations for 23 tumor suppressors and oncogenes. HPV status, p16 status, radiosensitivity data, and hypoxia data are summarized from all cell lines. We detected HPV transcripts in five cell lines, all of which overexpressed p16. One HPV negative cell line was also p16 positive. We detected mutations in *KIT* (SCCNij185), *PIK3CA* (SCCNij185), and *CDKN2A* (UT-SCC-5 and UT-SCC-38). *TP53* mutations were the most frequent, occurring in 16/22 cell lines. HPV infection and *TP53* mutations were almost mutually exclusive, with the exception of 93-VU-147T. The cell lines exhibited a wide range of sensitivities towards hypoxia and irradiation. Here, we provide a description of a set of frequently used HNSCC cell lines with diverse characteristics as found in HNSCC patients.

## 1. Introduction

Head and neck squamous cell carcinomas (HNSCC) are a malignant disease occurring in the mucosal regions of the oral cavity, hypopharynx, oropharynx, larynx, and other sites in the upper respiratory tract. It is a heterogeneous group of malignancies that, depending on disease type and location, has a relatively poor prognosis [1,2]. Five-year survival rates in oropharyngeal cancer are approximately 65% [1,2]. Treatment of HNSCC consists of a combination of surgery, platinum-based chemotherapy, radiotherapy, and—more recently—PD1 immune checkpoint inhibitors for recurrent HNSCC [2,3,4,5]. The most well-described risk factors for HNSCC include smoking, alcohol consumption, and high-risk human papillomavirus (HPV) infection [1,2,3,4,5]. Particularly in oropharyngeal cancer, the prevalence of HPV infection is relatively high with up to 81.4% of the patients infected with the virus [6,7]. Several studies have shown that patients with HPV positive tumors, and—more specifically—subsequent p16 overexpression, have an improved prognosis [1,2,5,8]. In these patients, there is superior locoregional control in response to radiotherapy, and overall- and event-free survival [1,2,5,8]. In order to identify mechanisms that influence sensitivity or resistance to treatment, molecular and cellular characterization of HPV positive and HPV negative cells has been widely employed [9]. In vitro as well as in vivo characterization of these mechanisms heavily depends on the use of HNSCC cell lines, and many have been distributed over the world to study the disease [9]. A little over 300 HNSCC cell lines of various tumor origins and patient characteristics such as age, sex, and ethnicity have been established [9]. A significant drawback of the widely available HNSCC cell lines and their distribution across the globe is that cross-contamination and mislabeling have led to contradictions in literature and incorrect conclusions drawn from experiments performed with the wrong cell lines [10]. Here, we provide genetic analysis of a set of HNSCC cell lines that are widely used, using short tandem repeat profiling. Additionally, we established a database-like compendium about multiple genetic, molecular, and cellular characteristics. These include cell morphology, mutation status of 23 tumor suppressor and oncogenes, HPV status, p16 status, and hypoxia and radiosensitivity. Together, this makes a useful tool for other researchers who wish to study different subsets of HNSCC cells.

## 2. Results

### 2.1. HNSCC Cell Line Panel Characteristics and Genotyping

We collected 22 HNSCC cell lines frequently used in in vitro and in vivo studies (Table 1). For SCCNij cell lines, patient characteristics were largely anonymized, aside from tumor stage, lesion type, and tumor location. All of the remaining 17 cell lines were from male HNSCC patients. Patients were on average 57 (±13.6) years old at time of the biopsy (Table 1 [11,12,13,14,15,16,17,18,19,20,21]). Out of 22 cell lines, 18 (81.8%) cell lines were derived from primary tumors, whereas 3 (13.6%) were derived from recurrences, and 1 cell line (4.5%) from a metastasis. The cell lines had various anatomical origins, with 9 cell lines (40.9%) originating from the oral cavity (tongue, floor of mouth), 11 cell lines (50%) from the larynx (supraglottic and glottic larynx), 1 cell line from the oropharynx (4.5%), and 1 cell line from the hypopharynx (4.5%). In this panel, 10 cell lines (45.5%) were derived from T1–T2 stage tumors, 11 cell lines from a T3–T4 stage tumor (50%), and of one cell line (4.5%) T stage was not available. 13 cell lines (59.1%) were from patients with N0 disease, 8 cell lines (36.4%) from patients with N1–2 stage disease, and of one cell line (4.5%) N stage was not available. Lastly, 18 cell lines (81.8%) were derived from M0 stage disease, whereas M stage information was lacking from all other cell lines (18.2%) (Table 1 [11,12,13,14,15,16,17,18,19,20,21]).

The short tandem repeat (STR) profiles of the UT-SCC-19A, UT-SCC-24A, UT-SCC-45, UM-SCC-6, UM-SCC-47, 93-VU-147T, UPCI: SCC090 and UPCI: SCC154 cell lines were identical to previously reported profiles (Table S1) [10,22,23,24,25,26]. The remaining profiles were found to be unique as compared to another and to the Cellosaurus database. Commonly, the amelogenin locus on the X and Y chromosomes is used for gender identification. However, loss of detectable Y chromosome occurs frequently in older male donors and cell lines. Therefore, an X genotype in cell lines does not confirm that the cell line and donor are female [27,28].

### 2.2. HNSCC Cell Line Morphology

**Figure 1:**
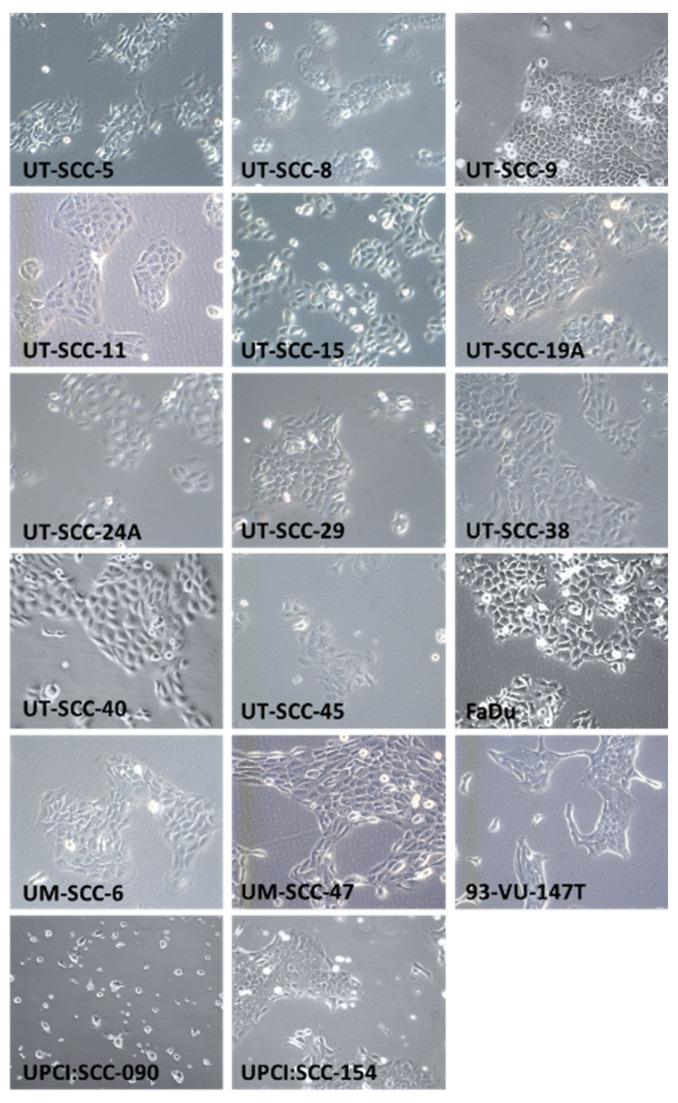
HNSCC cell line morphology. Cells were grown in T75 culture flasks for 2–3 days. Pictures represent seventeen HNSCC cell lines at 100× magnification using a phase-contrast microscope. SSC-Nij cell-lines do not grow in culture but only as xenografts in vivo; H&E stainings of these xenografts are shown in Supplementary Figure S1.

In addition to genetic characterization, information about cellular morphology is important to establish the authenticity of a cell line. For example, prolonged passaging and exposure to different culture media could potentially lead to changes in morphology, polarity, epithelial-mesenchymal transition, and therefore cellular behavior [29,30]. In order to document the morphology of our HNSCC cell line panel, we imaged each cell line at 100× magnification (Figure 1). Additionally, H&E sections of xenografts of the SSC-Nij cell lines, full width scans at 10× magnification, are shown in Supplementary Figure S1.

Furthermore, from a radiobiological perspective, the colony-forming properties of these cell lines is of particular interest, as the colony-forming assay remains currently the gold standard for radiosensitivity experiments [31]. UT-SCC-5, UT-SCC-8, UT-SCC-9, UT-SCC-11, UT-SCC-19A, UT-SCC-29, UT-SCC-38, UT-SCC-40 and FaDu demonstrate excellent colony-forming abilities and produce tightly packed, round colonies (Supplementary Figure S2A). UT-SCC-15, UT-SCC-45, and UM-SCC-6 cells form colonies, yet are relatively less tightly packed, and show a little more diffuse pattern (Supplementary Figure S2B). Lastly, UM-SCC-47, 93-VU-147T, UPCI:SCC090, and UPCI:SCC-154 are able to form colonies, yet either form very spread out, irregularly shaped colonies (UM-SCC-47, UPCI:SCC154, and 93-VU-147T (Supplementary Figure S2C)), or very dense, small colonies (UT-SCC-8 (Supplementary Figure S2D)).

### 2.3. Mutations in HNSCC Cell Lines

We characterized the presence of mutations in well-known oncogenes and tumor suppressor genes using single molecule Molecular Inversion Probe (smMIP) sequencing of 23 oncogenes and tumor suppressor genes. This method was previously established to test for clinical mutations in patient-derived samples and covers 41 hotspot regions required for cancer diagnoses and/or predictive diagnostics (Table S2) [32].

**Figure 2:**
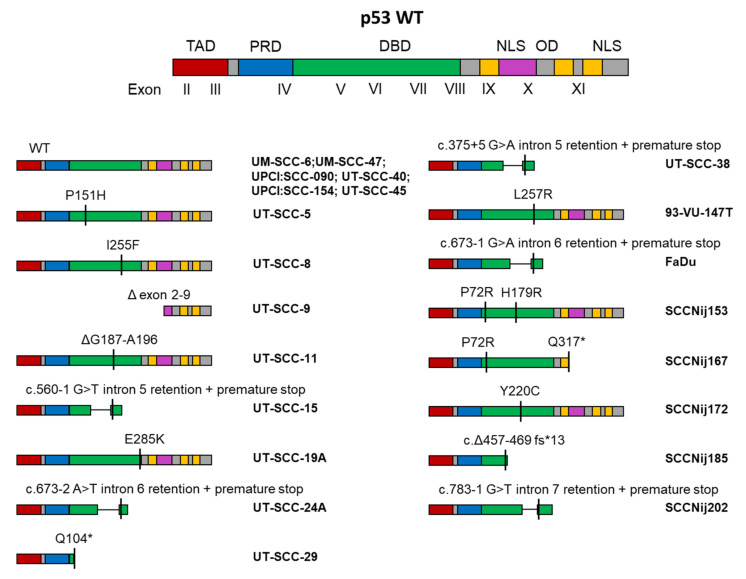
TP53 mutations in HNSCC cell lines. TP53 wildtype (WT) transcript is shown including exon locations and functional domains. For every detected TP53 mutation, the consequence for the TP53 transcript is shown for each cell line. TAD: transactivation domain; PRD: proline-rich domain; DBD: DNA binding domain; NLS: nuclear localization sequence; OD: oligomerization domain. * (asterisk) = translation termination (stop) codon.

In our HNSCC cell line panel we detected a number of mutations, mostly in the TP53 gene. Strikingly, all of the TP53 mutations that were detected were localized in the DNA and/or rendered a significant part of the DNA binding domain (DBD) dysfunctional through frameshifts, premature stop codons, or inappropriate intron retention (Figure 2). In four cell lines a mutation was detected in addition to the TP53 mutation (Table S3). The CDK2NA gene was mutated in both UT-SCC-5 and UT-SCC-38. In UT-SCC-5, a deletion in the CDK2NA gene resulted in a frameshift and premature stop codon (c.331_352del, p.Gly111Leufs * 28), whereas in UT-SCC-38, a splice mutation was detected (c.151-1G > T). Furthermore, in SCCNij185 we found a mutation in KIT (c.2122C > A, p.His708Asn). Lastly, a mutation in PIK3CA (c.1633G > A, p.Glu545Lys) was found in SCCNij185. Previous reports also demonstrated mutations in HRAS in 93-VU-147T (c.322G > T; p. Asp108Tyr), a CDKN2A deletion in FaDu, and NOTCH1 mutations in UM-SCC-47 (c.574G > T; p.Gly192 *) and UT-SCC-45 (c.214G > A p.Gly72Arg) [33].

The mutations in the TP53 gene, or lack thereof, were found to be identical to the original reports for UT-SCC-5 [34], UT-SCC-8 [34], UT-SCC-9 [34], UT-SCC-11 [13], UT-SCC-15 [35], UT-SCC-19A [13], UT-SCC-24A [36], UT-SCC-29 [13], UT-SCC-45 [23], FaDu [13], UM-SCC-6 [37], UM-SCC-47 [37], 94-VU-147T [33], UPCI:SCC090 [18,38], and UPCI:SCC154 [18]. To the best of our knowledge, the TP53 mutation status of UT-SCC-38 has not yet previously been tested, so no comparison was possible. We found a discrepancy between the original report and our data on the mutation status of UT-SCC-40. UT-SCC-40 was previously reported to have a TP53 mutation (c.637C > T p.Arg213 *), yet we did not detect any [13]. Interestingly, a later report showed a lack of p53 protein in UT-SCC-40 [39]. This could potentially indicate the presence of wildtype p53, as it is under normal circumstances actively degraded by MDM2, and mutated p53 is more often detectable as a (truncated) protein [40]. However, we cannot exclude the possibility that our UT-SCC-40 cell line is not the original UT-SCC-40 cell line we believe it to be. Furthermore, this is the first time the TP53 mutation status of the SCCNij cell lines has been determined, and can therefore not be compared to any other reports.

### 2.4. HPV and p16 Status

**Figure 3:**
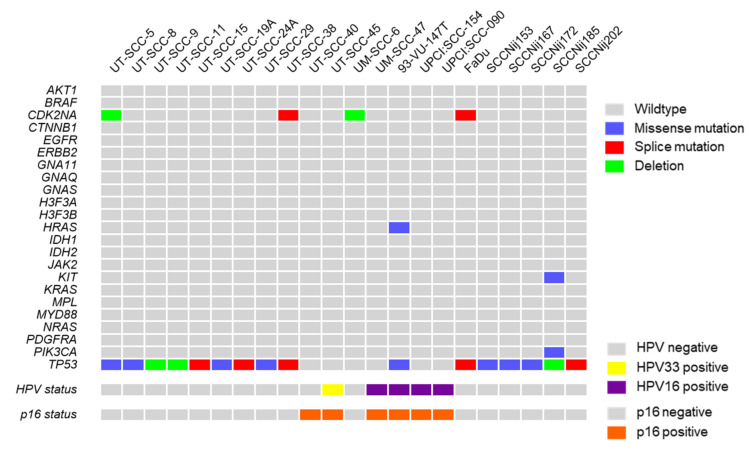
HNSCC cell line panel mutation, human papillomavirus (HPV), and p16 status. Shown are 22 cell lines and their mutation status of 23 tumor suppressors and oncogenes. In addition, HPV and p16 status are shown.

HPV status and p16 status have been shown to be important factors that determine how well tumors and patients respond to treatment. Characterization and documentation of these factors is therefore important. While HPV status and p16 status are commonly used interchangeably in literature, they have different effects on treatment outcome. We previously reported the p16 and HPV status and summarize them here (Figure 3). Previously, we demonstrated that UT-SCC-45 was infected with HPV33, and UPCI:SCC090, UPCI:SCC154, 93-VU-147T, and UM-SCC-47 were infected with HPV16, whereas we did not detect any HPV in other HNSCC cell lines [21,41]. In all the HPV positive cell lines (UT-SCC-45, UM-SCC-47, UPCI:SCC090, UPCI:SCC154, 93-VU-147T) p16 was overexpressed, but also in the HPV negative UT-SCC-40 cell line [42]. Interestingly, previous reports have shown that HPV positive HNSCC do not always have overexpressed p16 [43]. These and our own findings stress the difference between HPV and p16 status.

### 2.5. Radiosensitivity and Hypoxia Sensitivity

A significant number of HNSCC cell lines in our panel have been used for in vitro and in vivo studies regarding mechanisms that confer radioresistance or radiosensitivity to cells. For a number of these cell lines, we have collected and summarized the available data on their relative radiosensitivity [21,41,44]. We reported both the original surviving fractions in response to 2, 4, 6, and 8 Gy, as well as the derivative D37 (dose required for 37% survival) as calculated using the linear quadratic model: SF = e^ − (αD + βD^2^). As hypoxia (low tissue oxygen concentrations, usually below 2% O_2_), and especially severe hypoxia (<0.1% O_2_) is one of the best described variables to negatively affect radiosensitivity, we also included cellular sensitivity to low oxygen tensions. In addition, we summarized the hypoxic fractions that have been reported to occur in in vivo xenografts of individual cell lines, as measured using the hypoxia-marker pimonidazole (Table S4). In both of these studies, image segmentation was used to create binary images of tumor-delineated immunofluorescent images where the fraction of positive pixels was used to determine the hypoxic fraction of the tumor area [21,44].

## 3. Discussion

In this study, we characterized a panel consisting of 22 unique HNSCC cell lines. We extensively tested for genetic identity as well as mutations in a set of tumor suppressors and oncogenes. In addition, we documented cellular morphology, HPV infection, and p16 status. Lastly, we reported cellular sensitivity to irradiation as well as hypoxia. Taken together, we have provided a HNSCC cell line database that may be used by other researchers to select the appropriate culture model for HNSCC.

Several other investigators have attempted to summarize and characterize the available HNSCC cell line models that are in circulation between universities and research institutes [10,13,22,23]. However, several of the cell lines we studied have not yet been previously characterized and documented. We compared the STR profiles of each cell line and compared them to previously published profiles [10,22,23,24,26] where possible. As the xenografted SSC-Nij cell tumors will always contain heterogeneous amounts of mouse tissues, and no information is available for the original human tumors they were derived from, we did not STR-profile these cell lines. All previously characterized cell lines [10,22,23,24,26] matched with the STR profiles we describe here. Furthermore, for the first time we published the STR profiles of UT-SCC-5, -8, -9, -11, -15, 2–9, -38, and -45, which were all unique profiles not matching any currently known cell lines.

The cell line panel described here spans both HPV negative and HPV positive subtypes, and includes radio- and hypoxia sensitivity, as well as the degree of hypoxia observed in xenograft studies. Taken together, this provides valuable information regarding the tumor microenvironment and intrinsic sensitivity to irradiation, both explicitly important factors that determine success of radiotherapy.

Regarding patient characteristics, our HNSCC cell line panel was relatively diverse in terms of TNM stage and anatomical location. Previous studies have shown HNSCC, and especially HPV positive HNSCC, is more prevalent in males than females [45]. In our HNSCC cell line panel all cell lines were derived from male patients [11,12,13,14,15,16,17,18,19,20,21], which is a factor that has to be taken into account when selecting for a diverse HNSCC cell line panel.

Cancer arises by accumulation of genetic mutations, both activating mutations in oncogenes, as well as inactivating mutations in tumor suppressor genes. Information about both activating and inactivating mutations becomes more important in the age of personalized medicine, as specific mutations are known to affect treatment response. For example, the presence of inactivating BRCA mutations in breast cancer predicts the sensitivity to PARP inhibitors [46,47]. Therefore, we characterized the presence of mutations in well-known oncogenes and tumor suppressor genes using smMIP sequencing of 23 oncogenes and tumor suppressor genes. Whereas not specific for HNSCC, this method was previously established to test for clinical mutations in patient-derived samples and covers 41 hotspot regions required for cancer diagnoses and/or predictive diagnostics.

We used a targeted sequencing approach to detect mutations in a previously validated panel of tumor suppressors and oncogenes. While this is not an exhaustive list, this panel contains clinically relevant variations found in a wide range of malignancies [32]. We found mutations in PIK3CA, KIT, CDKN2A, but mostly in TP53 (72.3% of all cell lines). This is unsurprising given that in HPV negative HNSCC, 86% of the tumors have been reported to bear TP53 mutations [48]. TP53 is one of the best described tumor suppressor genes, and acts as a transcription factor linked to DNA damage response signalling, G1-S arrest, and apoptosis [49]. The TP53 gene encodes the p53 protein, and is highly conserved throughout evolution and species [49]. It is located on human chromosome 17p13.1, and consists of 11 exons. Exon 1 is non-coding, whereas exons 2 to 11 are translated into the p53 protein [49]. Multiple isoforms of p53 have been reported, which are achieved via alternative splicing in exon 9 [49]. The p53 protein consists of several functional domains, starting at the N-terminus with a transactivation domain, followed by a proline-rich domain, a DNA binding domain (DBD), nuclear localization sequence, oligomerization domain, and, finally, another nuclear localization sequence [49]. Importantly, most of the described pathogenic mutations in TP53 are located in the DNA binding domain, which usually inactivates p53′s ability to interact with DNA and therefore abrogates its transcriptional activity [50]. Other relatively frequent mutations found in HNSCC, but that were not part of our smMIP panel, were reported in NOTCH1 and KMT2D [48].

Interestingly, HPV status and *TP53* mutations were almost mutually exclusive, except in the 93-VU-147T cell line. This was also reflected in the HNSCC patient population, where HPV positive tumors almost never harbored *TP53* mutations [48,51]. All the *TP53* mutations we detected in 16 cell lines lead to either abolished or defective *TP53* transcripts at the level of the DNA binding domain. This has previously been reported to be the hotspot of *TP53* mutations in HNSCC and we confirmed this in our cell line panel [52]. Despite our targeted sequencing approach, we were unable to detect mutations in *CDKN2A* in the FaDu and UM-SCC-6 cell lines, whereas previous reports have demonstrated that they carry a splice mutation and deletion, respectively [33]. Since STR profiling demonstrated that these cell lines were correctly identified, we believe the cell line to be FaDu regardless. One possible explanation might be that, despite using a validated method, the sequencing depth at that specific location may have been insufficient to detect the mutation.

Additionally, we collected data regarding HPV and p16 status. These are often used interchangeably, yet may orchestrate different responses in terms of radiosensitivity [45]. All HPV positive cell lines overexpressed p16, which is a canonical feedback mechanism attributed to the effects of E7 in the cell [53]. As a result of E7 expression, RB is primed for proteasomal degradation, which in turn liberates the E2F transcription factor. One of the transcription targets of E2F is *CDK2NA*, which encodes the p16 protein, completing the feedback loop [53]. We found that UT-SCC-40 is an HPV negative cell line that overexpresses p16, which is not caused by any *CDKN2A* activating mutations [42]. It remains unclear why UT-SCC-40 overexpresses p16, yet it does belong to one of the most radiosensitive cell lines in our panel, alongside the HPV positive cell lines. This further strengthens the notion that mainly p16, and not HPV status, is a main determinant of radiosensitivity.

The radiosensitivity data we collected confirm that HPV positive HNSCC cell lines are more radiosensitive than HPV negative cell lines, and are in agreement with previous publications [41,42,54,55,56]. To the best of our knowledge no other reports exist that describe the sensitivity of these HNSCC cell lines to hypoxia.

## 4. Materials and Methods

### 4.1. Cell Culture

UT-SCC-5, UT-SCC-8, UT-SCC-9, UT-SCC-11, UT-SCC-15, UT-SCC-19A, UT-SCC-24A, UT-SCC-29, UT-SCC-38, UT-SCC-40, and UT-SCC-45 cell lines were kindly provided by Prof. Grenman, University of Turku, Turku, Finland. UM-SCC-6 and UM-SCC-47 were kindly provided by Dr. Carey, University of Pittsburgh, Pittsburgh, PA, USA). FaDu cells were kindly provided by Michael Baumann (German Cancer Research Center, Heidelberg, Germany). 93-VU-147T was kindly provided by Dr. Dorsman, Amsterdam University Medical Center, Amsterdam, the Netherlands). UPCI:SCC090 and UPCI:SCC154 were purchased at DSMZ. All cell lines were cultured in DMEM medium (Gibco, Invitrogen, CA, USA) supplemented with 4.5 g/L glucose, GlutaMAX, 10% FBS, 100 u/mL penicillin/streptomycin, non-essential amino acids (Gibco), HEPES (Gibco), and sodium pyruvate (Gibco). The SCCNij lines were derived from patient biopsies obtained between 1996 and 2006 at the Radboud university medical center (Nijmegen, the Netherlands) and passaged as xenograft models in BALB/c *nu/nu* mice [21,44]. Cell line characteristics are described in Table 1.

### 4.2. Microscopy

All cells (except SCCNij) were grown to approximately 70% confluency. Phase-contrast images were taken using a Zeiss AXIO microscope and Zeiss AxioCam ICc5 camera (Zeiss, Oberkochen, Germany) at 100× magnification. Software used was Zen 2012 (Blue edition).

### 4.3. Genomic DNA Extraction

HNSCC cell lines (aside from SCCNij) were cultured to approximately 70% confluency and then harvested by cell scraping. Genomic DNA extraction was performed by using a genomic DNA extraction kit (Norgen Biotek, Thorold, ON, Canada). Cells were lysed using a digestion buffer, followed by an RNAse A treatment (10 kU per sample). Proteinase K treatment was performed for approximately 1 h at 55 °C. Samples were washed with 100% ethanol and transferred to a binding column and centrifuged at >5000× *g* for 3 min. Lysates bound to the column were then repeatedly washed with 100% ethanol and subsequently eluted into a nucleic acid free tube. gDNA was quantified by spectrophotometric analysis. For SCCNij tumors, ex vivo frozen chunks were thawed and homogenized using sonification. Genomic DNA was then isolated in a similar manner as described above.

### 4.4. Mutation Analysis

Mutation analysis of a panel of cancer-related oncogenes and tumor suppressor genes was performed by single molecule Molecular Inversion Probe (smMIP) sequencing. Sequencing was performed on 100 ng genomic DNA at the department of Pathology, Radboud University Medical Center (Nijmegen, the Netherlands), according to a previously described protocol [32]. Targeted genes are described in Table S3.

### 4.5. STR Profiling

Short tandem repeat (STR) profiling was performed by Eurofins genomics (Ebersberg, Germany) on gDNA extracted from the HNSCC cell lines. STR analysis was performed for 16 markers, including CSF1PO, D2S1338, D3S1358, D5S818, D7S820, D8S1179, D13S317, D16S539, D18S51, D19S433, D21S11, FGA, TH01, TPOX, vWA, and amelogenin. Where possible, STR profiles were compared to previously published consensus profiles, as well as the Cellosaurus database of existing STR profiles. STR analyses were not performed for the SSCNij lines, as these are grown in mice, which would result in mixed STR profiles.

### 4.6. Colony Forming Assays

Radiosensitivity data were acquired by colony-forming assays. Data on all cell lines were available from previously published work, except for UT-SCC-9 and FaDu [41,42]. In brief, cells were plated as single cell suspensions and incubated overnight. Single dose irradiation was delivered using a 320 kV XRAD irradiator (RPS Services Limited, Surrey, UK) at a dose rate of 3.1 Gy/min. Medium was refreshed 24 h after IR and cells were left to form colonies for 8–14 days. Colonies were fixed and stained by crystal violet staining (50% methanol, 20% ethanol, 30% water, 5 mg/mL crystal violet).

## 5. Conclusions

Taken together, we have established an informative HNSCC cell line database. We believe that these cell lines are suitable models to study HNSCC disease in vitro and in vivo and may provide a valuable tool for investigators wishing to study the relation between hypoxia and radiosensitivity in HNSCC.

## Figures and Tables

**Table 1:** Head and neck squamous cell carcinoma (HNSCC) cell lines: patient characteristics.

Cell Line	Age	Sex	TNM Status	Primary Tumor Location	Lesion Type	Reference
UT-SCC-5	58	Male	T1N1M0	Tongue	Primary	[11]
UT-SCC-8	42	Male	T2N0M0	Supraglottic larynx	Primary	[11]
UT-SCC-9	81	Male	T2N0M0	Glottic larynx	Primary	[11]
UT-SCC-11	58	Male	T1N0M0	Glottic larynx	Recurrence	[12]
UT-SCC-15	51	Male	T1N0M0	Tongue	Recurrence	[13]
UT-SCC-19A	44	Male	T4N0M0	Glottic larynx	Primary	[14]
UT-SCC-24A	41	Male	T2N0M0	Tongue	Primary	[11]
UT-SCC-29	82	Male	T2N0M0	Glottic larynx	Primary	[11,14]
UT-SCC-38	66	Male	T2N0M0	Glottic larynx	Primary	[13]
UT-SCC-40	65	Male	T3N0M0	Tongue	Primary	[13]
UT-SCC-45	76	Male	T3N1M0	Floor of mouth	Primary	[13]
UM-SCC-6	37	Male	T2N0M0	Oropharynx	Primary	[15]
UM-SCC-47	53	Male	T3N1M0	Tongue	Primary	[16]
93-VU-147T	58	Male	T4N2	Floor of mouth	Primary	[17]
UPCI:SCC090	46	Male	T2N0	Base of tongue	Recurrence	[18]
UPCI:SCC154	54	Male	T4N2	Tongue	Primary	[18]
FaDu	56	Male	NA	Hypopharynx	Metastasis	[19,20]
SCCNij153	Unknown	Unknown	T3N2M0	Supraglottic larynx	Primary	[21]
SCCNij167	Unknown	Unknown	T4N2M0	Supraglottic larynx	Primary	[21]
SCCNij172	Unknown	Unknown	T4N0M0	Supraglottic larynx	Primary	[21]
SCCNij185	Unknown	Unknown	T4N1M0	Supraglottic larynx	Primary	[21]
SCCNij202	Unknown	Unknown	T4N0M0	Supraglottic larynx	Primary	[21]

## Data Availability

All data is available upon request.

## Supplementary Materials

The following are available online at https://www.mdpi.com/2072-6694/13/5/1069/s1. Figure S1. H&E stainings of SSC-Nij cell-lines grown as xenografts in vivo; Figure S2. Examples of different types of colony forming abilities found in the HNSCC cell lines; Table S1. STR profile of HNSCC cell lines; Table S2. Panel of sequenced mutation hotspots across 23 genes; Table S3. Mutations detected in HNSCC cell lines; Table S4. Radiosensitivity and hypoxia sensitivity HNSCC cell line panel.
