# Supplementary material for: Genotyping and Characterization of HPV Status, Hypoxia, and Radiosensitivity in 22 Head and Neck Cancer Cell Lines
Source: Cancers (Basel). 2021 Mar 3;13(5):1069. doi: 10.3390/cancers13051069 (PMC7959143; doi:10.3390/cancers13051069)
Supplement: Supplementary file 1 [file cancers-13-01069-s001.pdf]

cancers MDPI

Supplementary Materials: Genotyping and characterization of HPV status, hypoxia, and radiosensitivity in 22 head and neck cancer cell lines

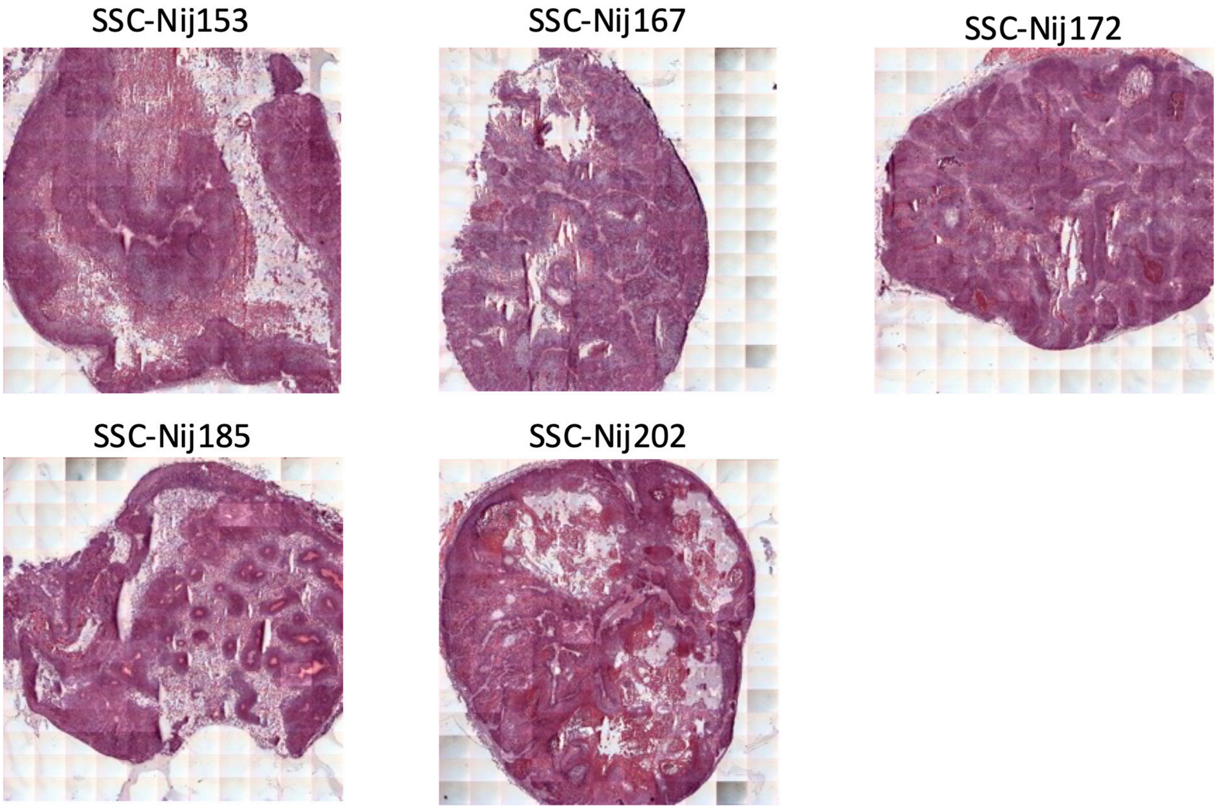

Figure S1. H&E stainings of SSC-Nij cell-lines grown as xenografts in vivo.

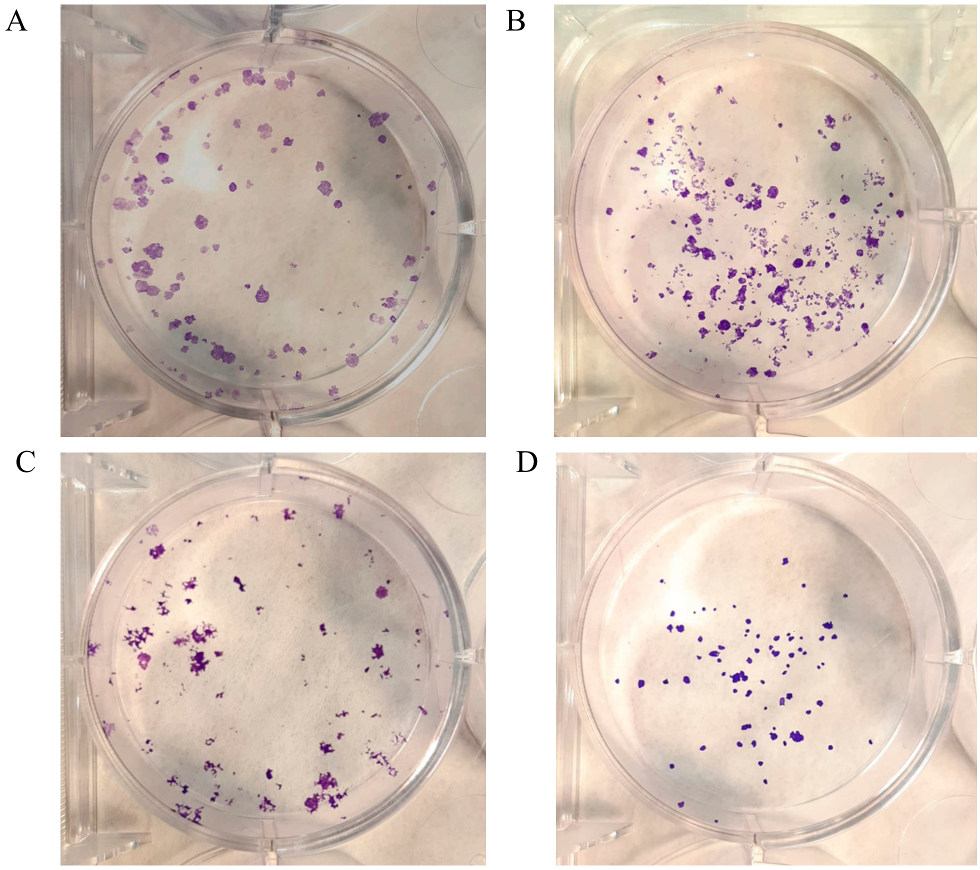

Figure S2. Examples of different types of colony forming abilities found in the HNSCC cell lines, giving either A) tightly packed, round colonies (i.e. UT-SCC-5, UT-SCC-8, UT-SCC-9, UT-SCC-11, UT-SCC-19A, UT-SCC-29, UT-SCC-38, UT-SCC-40, FaDu), B) more diffuse pattern (UT-SCC-15, UT-SCC-45, UM-SCC-6), C) irregularly shaped colonies (UM-SCC-47 , UPCI:SCC154, 93-VU-147T) or D) very dense, small colonies (UT-SCC-8).

Table S1. STR profile of HNSCC cell lines.

Cell line	STR profile markers								
	D8S1179	D21S11	D7S820	CSF1PO	D3S1358	TH01	D13S317	D16S539	D2S1338
UT-SCC-5	13, 14	30, 30	10, 10	10, 10	16, 16	7, 9.3	9, 12	11, 11	17, 22
UT-SCC-8	13, 14	30, 31.2	10, 10	10, 11	15, 15	9.3, 9.3	8, 14	12, 12	19, 25
UT-SCC-9	12, 13	29, 31.2	8, 12	10, 11	15, 15	7, 9.3	12, 12	11, 12	25, 25
UT-SCC-11	12, 12	28, 28	9, 11	10, 10	17, 17	6, 9	9, 11	9, 11	19, 20
UT-SCC-15	13, 13	28, 28	8, 9	12, 15	15, 15	6, 8	11, 11	11, 12	19, 19
UT-SCC-19A	10, 14	30, 32.2	12, 12	12, 12	15, 15	6, 6	11, 12, 13	12, 13	19, 19
UT-SCC-24A	13, 15	29, 30	11, 11	13, 13	16, 16	9.3, 9.3	12, 14	11, 11	20, 20
UT-SCC-29	10, 13	31, 31	8, 1	12, 12	16, 18	9.3, 9.2	11, 11	11, 11	17, 20
UT-SCC-38	13, 13	32.2, 32.2	8, 10	9, 12	16, 16	9.3, 9.3	9, 12	12, 14	20, 20
UT-SCC-40	12, 14	29, 31.2	8, 10	11, 13	17, 17	7, 9	11, 12	12, 12	17, 25
UT-SCC-45	13, 18	31.2, 31.2	11, 12	10, 12	15, 15	7, 9.3	11, 14	9, 13	19, 19
UM-SCC-6	11, 14	28, 35.1	10, 11	10, 10	15, 15	6, 9.3	13, 13	9, 12	19, 20
UM-SCC-47	15, 15	29, 30	11, 11	11, 13	15, 15	7, 9.3	8, 9, 11	8, 13	25, 25
93-VU-147T	13, 15	31, 31	10, 11	11, 12	15, 17	7, 9	12, 12	9, 11	20, 24
UPCI:SCC090	12, 12	29, 31	9, 10	11, 12	14, 14	7, 7	11, 11	12, 13	22, 22
UPCI:SCC154	12, 12	28, 29	9, 10	10, 12	16, 16	7, 7	9, 12	13, 13	25, 25
FaDu	13, 13	31.2, 31.2	11, 12	12, 12	17, 17	8, 8	8, 9	11, 11	19, 19

Table S1. Continued. STR profile of HNSCC cell lines.

Cell line	STR profile markers							
	D19S433	vWA	TPOX	D18S51	AMEL*	D5S818	FGA	Comparison to consensus	Reference
UT-SCC-5	15, 15	15, 16	8, 12	18, 18	X, Y	10, 10	22, 23	NA	na
UT-SCC-8	13, 13	14, 16	8, 8	13, 13	X	10, 11	21, 21	NA	na
UT-SCC-9	15, 15	14, 16	8, 8	18, 20	X, Y	13, 14	20, 20	NA	na
UT-SCC-11	13, 14	16, 17	9, 11	15, 17	X, Y	13, 13	22, 23	NA	na
UT-SCC-15	15, 15.2	17, 18	8, 9	12, 14	X	12, 13	25, 25	NA	na
UT-SCC-19A	13.2, 18.2	14, 19	8, 10	16, 16	X	12, 12	24, 24	Identical	[1]
UT-SCC-24A	12, 14.2	16, 18	11, 11	18, 18	X	10, 10	19, 22	Identical	[1]
UT-SCC-29	14, 14	17, 18	8, 8	15, 15	X, Y	11, 11	21, 21	NA	na
UT-SCC-38	13, 15	18, 20	11, 11	15, 22	X, Y	11, 11	22, 22	NA	na
UT-SCC-40	12, 12	17, 19	8, 12	13, 13	X, Y	13, 13	22, 22	NA	na
UT-SCC-45	14, 15	18, 20	11, 11	15, 15	X	11, 12	21, 23	Identical	[2]
UM-SCC-6	11, 14	15, 16	11, 11	11, 19	X, Y	12, 12	23, 23	Identical	[4,5]
UM-SCC-47	14, 15	18, 18	10, 11	18, 18	X, Y	11, 12	23, 25	Identical	[2–5]
93-VU-147T	14, 15	18, 18	9, 11	16, 16	X	11, 12	22, 22	Identical	[2,5]
UPCI:SCC090	13, 13	17, 17	8, 8	14, 18	X, Y	11, 12	20, 20	Identical	[2,5]
UPCI:SCC154	15.2, 16	17, 17	8, 9	15, 15	X, Y	11, 12	20, 24	Identical	[2]
FaDu	14, 16	15, 17, 18	11, 11	16, 16	ND	12, 12	21, 23	Identical	[4,6]

* Due to frequent loss of Y chromosomes in cell lines, amelogenin (AMEL) is not used for comparison to consensus. NA: not available. na: not applicable. ND: Not detected.

Table S2. Panel of sequenced mutation hotspots across 23 genes.

Gene	Exon	Targeted codons
AKT1	03	E17
BRAF	15	D594-K601
CDKN2A	01a, 01b, 02, 03	>95% of all coding sequences and splicing sequences
CTNNB1	03	D32-S45
EGFR	12, 18-21	S492, E709, exon 19, exon 20, L858-L861
ERBB2 (HER2)	20	Y772-Y781
GNA11	04, 05	R183, Q209
GNAQ	04, 05	R183, Q209
GNAS	08, 09	R201, Q227
H3F3A	02	K28, G35
H3F3B	02	K37
HRAS	02, 03	G12, G13, H27, A59, Q61
IDH1	04	R132
IDH2	04	R140, R172
JAK2	14	V617
KIT	08, 09, 11, 13, 14, 17	Exon 8, 9, 11, K642-N655, exon 14, D816-Y823
KRAS	02, 03, 04	G12, G13, A59, Q61, K117, A146
MPL	10	W515
MYD88	05	L265
NRAS	02, 03, 04	G12, G13, A59, Q61, K117, A146
PDGFRA	12, 14, 18	Exon 12, 14, V824-D842
PIK3CA	10, 21	E542-Q546, M1043-G1049
TP53	01-11	>95% of all coding sequences and splicing sequences

Table S3. Mutations detected in HNSCC cell lines.

**Cell line**	***TP53* mutation**	**Additional mutations**
UT-SCC-5	c.452_453delinsAT; p.Pro151His	*CDKN2A* c.331_352del; p.Gly111Leufs*28
UT-SCC-8	c.763A>T; p.Ile255Phe	
UT-SCC-9	Δ exon 2-9	
UT-SCC-11	c.560_589del; p.Gly187_Arg196del	
UT-SCC-15	c.560-1G>T	
UT-SCC-19A	c.853G>A; p.Glu285Lys	
UT-SCC-24A	c.673-2A>T	
UT-SCC-29	c.310C>T; p.Gln104*	
UT-SCC-38	c.375+5G>A	*CDKN2A* c.151-1G>T
UT-SCC-40		
UT-SCC-45		*NOTCH1* p.Gly72Arg (c.214G>A) [7]
FaDu	c.673-1G>A	*CDKN2A* c.151-1G>T; *SMAD4* c.1_1659del1659 [7]
UM-SCC-6		*CDKN2A* deletion [7]
UM-SCC-47		*NOTCH1* p.Gly192Ter (c.574G>T) [7]
93-VU-147T	c.770T>G; p.Leu257Arg	*HRAS* p.Asp108Tyr (c.322G>T) [7]
UPCI:SCC154		
UPCI:SCC090		
SCCNij153	c.215C>G p.Pro72Arg<br>c.536A>G; p.His179Arg	
SCCNij167	c.215C>G p.Pro72Arg<br>c.949C>T; p.Gln317*.	
SCCNij172	c.659A>G; p.Tyr220Cys	
SCCNij185	c.457_469del; p.Pro153Serfs*13	*KIT* c.2122C>A, p.His708Asn<br>*PIK3CA* c.1633G>A, p.Glu545Lys
SCCNij202	c.783-1G>T	

c.673: coding DNA sequence change at position 673; p.: amino acid change; G>A: guanine to adenine substitution; Pro151His; amino acid 151 change from proline to histidine; -1G>T: substitution in splice site; del: deletion; ins: insertion; fs: frameshift; *: stop codon.

Table S4. Radiosensitivity and hypoxia sensitivity HNSCC cell line panel.

Cell line	SF2	SF4	SF6	SF8	α	β	D37 (Gy)	SF_{hypox}	Hypoxic fraction	Reference
UT-SCC-5	0.570	0.325	0.141	0.0491	0.203	0.0210	3.56	0.768	0.17 ± 0.05	[8,9]
UT-SCC-8	0.514	0.122	0.0199	0.0034	0.323	0.0500	2.28	0.790	0.10 ± 0.02	[8,9]
UT-SCC-9	0.499	0.250	0.165	0.0881	0.361	-0.0077	2.94	NA	NA	This paper
UT-SCC-11	0.525	0.180	0.0608	0.0126	0.273	0.0340	2.72	0.896	NA	[8]
UT-SCC-15	0.828	0.594	0.258	0.0792	-0.033	0.0430	5.17	NA	0.13	[8,9]
UT-SCC-19A	0.665	0.335	0.168	0.0684	0.190	0.0180	3.83	0.667	NA	[8]
UT-SCC-24A	0.434	0.143	0.0274	0.0043	0.313	0.0470	2.36	0.913	NA	[8]
UT-SCC-29	0.447	0.141	0.0488	0.0081	0.329	0.0331	2.43	0.944	0.10 ± 0.06	[8,9]
UT-SCC-38	0.402	0.121	0.0257	0.0066	0.443	0.0240	2.02	0.881	0.28 ± 0.07	[8,9]
UT-SCC-40	0.320	0.045	0.0217	0.0043	0.670	0.0006	1.48	0.492	NA	[8]
UT-SCC-45	0.319	0.064	0.0124	0.0008	0.424	0.0570	1.87	0.869	0.21 ± 0.04	[8,9]
FaDu	0.489	0.206	0.111	0.0500	0.381	-0.0011	2.63	NA	0.2 ± 0.05	This paper, [8]
UM-SCC-6	0.494	0.118	0.0124	0.0005	0.114	0.105	2.58	0.877	NA	[8]
UM-SCC-47	0.312	0.037	0.0022	0.0005	0.705	0.0330	1.33	0.669	NA	[8]
93-VU-147T	0.284	0.071	0.0157	0.0049	0.668	0.0004	1.49	0.595	NA	[8]
UPCI:SCC154	0.323	0.103	0.0095	0.0005	0.277	0.0830	2.17	NA	NA	[8]
SCCNij153	NA	NA	NA	NA	NA	NA	NA	NA	0.29 ± 0.10	[10]
SCCNij167	NA	NA	NA	NA	NA	NA	NA	NA	0.28 ± 0.14	[10]
SCCNij172	NA	NA	NA	NA	NA	NA	NA	NA	0.18 ± 0.14	[10]
SCCNij185	NA	NA	NA	NA	NA	NA	NA	NA	0.17 ± 0.10	[10]
SCCNij202	NA	NA	NA	NA	NA	NA	NA	NA	0.34 ± 0.21	[10]

Data are presented as mean ± SD. **SF2:** surviving fraction at 2 Gy; **SF4:** Surviving fraction at 4 Gy; **SF6:** Surviving fraction at 6 Gy; **SF8:** surviving fraction at 8 Gy; **α:** linear component of the linear quadratic model; **β:** quadratic component of the linear quadratic model; **SF_{hypox}** surviving fraction after 48 hours at 0.1% O_2; NA: not available.

references

1. Jamieson, S.M.; Tsai, P.; Kondratyev, M.K.; Budhani, P.; Liu, A.; Senzer, N.N.; Chiorean, E.G.; Jalal, S.I.; Nemunaitis, J.J.; Kee, D.; et al. Evofosfamide for the treatment of human papillomavirus-negative head and neck squamous cell carcinoma. *JCI Insight* **2018**, *3*, doi:10.1172/jci.insight.122204.
2. Kalu, N.N.; Mazumdar, T.; Peng, S.; Shen, L.; Sambandam, V.; Rao, X.; Xi, Y.; Li, L.; Qi, Y.; Gleber-Netto, F.O.; et al. Genomic characterization of human papillomavirus-positive and -negative human squamous cell cancer cell lines. *Oncotarget* **2017**, *8*, 86369–86383, doi:10.18632/oncotarget.21174.
3. Brenner, J.C.; Graham, M.P.; Kumar, B.; Saunders, L.M.; Kupfer, R.; Lyons, R.H.; Bradford, C.R.; Carey, T.E. Genotyping of 73 UM-SCC head and neck squamous cell carcinoma cell lines. *Head Neck* **2010**, *32*, 417–426, doi:10.1002/hed.21198.
4. Zhao, M.; Sano, D.; Pickering, C.R.; Jasser, S.A.; Henderson, Y.C.; Clayman, G.L.; Sturgis, E.M.; Ow, T.J.; Lotan, R.; Carey, T.E.; et al. Assembly and initial characterization of a panel of 85 genomically validated cell lines from diverse head and neck tumor sites. *Clin. Cancer Res. Off. J. Am. Assoc. Cancer Res.* **2011**, *17*, 7248–7264, doi:10.1158/1078-0432.CCR-11-0690.
5. Martin, D.; Abba, M.C.; Molinolo, A.A.; Vitale-Cross, L.; Wang, Z.; Zaida, M.; Delic, N.C.; Samuels, Y.; Lyons, J.G.; Gutkind, J.S. The head and neck cancer cell oncogenome: A platform for the development of precision molecular therapies. *Oncotarget* **2014**, *5*, 8906–8923, doi:10.18632/oncotarget.2417.
6. Yu, M.; Selvaraj, S.K.; Liang-Chu, M.M.; Aghajani, S.; Busse, M.; Yuan, J.; Lee, G.; Peale, F.; Klijn, C.; Bourgon, R.; et al. A resource for cell line authentication, annotation and quality control. *Nature* **2015**, *520*, 307–311, doi:10.1038/nature14397.
7. van Harten, A.M.; Poell, J.B.; Buijze, M.; Brink, A.; Wells, S.I.; Rene Leemans, C.; Wolthuis, R.M.F.; Brakenhoff, R.H. Characterization of a head and neck cancer-derived cell line panel confirms the distinct TP53-proficient copy number-silent subclass. *Oral Oncol.* **2019**, *98*, 53–61, doi:10.1016/j.oraloncology.2019.09.004.
8. Gottgens, E.L.; Bussink, J.; Leszczynska, K.B.; Peters, H.; Span, P.N.; Hammond, E.M. Inhibition of CDK4/CDK6 Enhances Radiosensitivity of HPV Negative Head and Neck Squamous Cell Carcinomas. *Int. J. Radiat. Oncol. Biol. Phys.* **2019**, *105*, 548–558, doi:10.1016/j.ijrobp.2019.06.2531.
9. Gillison, M.L.; Chaturvedi, A.K.; Anderson, W.F.; Fakhry, C. Epidemiology of Human Papillomavirus-Positive Head and Neck Squamous Cell Carcinoma. *J. Clin. Oncol. Off. J. Am. Soc. Clin. Oncol.* **2015**, *33*, 3235–3242, doi:10.1200/JCO.2015.61.6995.
10. Hoeben, B.A.; Starmans, M.H.; Leijenaar, R.T.; Dubois, L.J.; van der Kogel, A.J.; Kaanders, J.H.; Boutros, P.C.; Lambin, P.; Bussink, J. Systematic analysis of 18F-FDG PET and metabolism, proliferation and hypoxia markers for classification of head and neck tumors. *BMC Cancer* **2014**, *14*, 130, doi:10.1186/1471-2407-14-130.

© 2021 by the authors. Submitted for possible open access publication under the terms and conditions of the Creative Commons Attribution (CC BY) license (http://creativecommons.org/licenses/by/4.0/).
